# Pre-hospital analgesia in pediatric trauma and critically ill patients: An analysis of a German air rescue service

**DOI:** 10.1186/s13049-023-01069-x

**Published:** 2023-01-28

**Authors:** Christine Eimer, Florian Reifferscheid, Philipp Jung, Marcus Rudolph, Tom Terboven, Florian Hoffmann, Ulf Lorenzen, Andrea Köser, Stephan Seewald

**Affiliations:** 1 Department of Anesthesiology and Intensive Care Medicine, University Medical Centre Schleswig-Holstein, Campus Kiel, Arnold-Heller Str. 3, 24105 Kiel, Germany; 2 Department of Pediatrics, German Air Rescue Service Association “DRF Luftrettung”, Filderstadt, Germany; 3 University Medical Centre Schleswig-Holstein, Campus Lübeck, Lübeck, Germany; 4 Department of Anesthesiology and Intensive Care Medicine, University Medical Centre Mannheim, Mannheim, Germany; 5 Department of Anaesthesiology and Critical Care Medicine, St. Josefskrankenhaus, Heidelberg, Germany; 6 Dr. Von Hauner University Children’s Hospital, Munich, Germany; 7 Department of Emergency Medicine, University Medical Centre Schleswig-Holstein, Kiel, Germany

**Keywords:** Pediatric trauma, Pre-hospital analgesia, Fentanyl, Ketamine, Pain scale, Numerical rating scale, Emergency medical service, Helicopter emergency service

## Abstract

**Background:**

Pain management in the pre-hospital setting remains a particular challenge for paramedics and emergency physicians, especially in children. This study evaluates the pre-hospital use and effect of analgesics in children with trauma or pain due to other reasons.

**Methods:**

This study is a retrospective analysis of the database of a German air rescue service and was conducted over a period of 9 years (2012–2020) to assess pain in general and whether patients with trauma pain due to other reasons received treatment with analgesics. We included all patients in the registry under the age of 16 years. Patients with a Glasgow Coma Scale of 3 at hospital admission and incomplete records were excluded. The intensity of pain was determined by the emergency physician on scene at arrival and hospital admission in a ten-point rating scale (0 = no pain). Effective pain reduction was analyzed.

**Results:**

Out of 227,458 cases, a total of 22,025 emergency cases involved pediatric patients aged 0–16 years. 20,405 cases were included in the study. 12,000 (58.8%) children had suffered a trauma, 8108 (39.7%) had pain due to other reasons and 297 (1.5%) had both. In total, 4,608 (38.4%) of the children with trauma were assessed having a numerical rating scale (NRS) > 4 at EMS arrival. These patients received mainly ketamine (34.5%) and the opioids fentanyl (38.7%) and piritramide (19.1%). The value on the NRS was significantly lower at admission to hospital (mean 1.9) compared with the EMS arrival (mean 6.9). In 4.9% the NRS at hospital admission was still > 4. 282 patients within the non-trauma group had a pre-hospital NRS of > 4. The pain therapy consisted of opioids (35.8%) and ketamine (2.8%). 28.4% patients in the non-trauma group received no pain medication. In 16.0% the NRS at hospital admission was still > 4.

**Conclusions:**

German emergency physicians achieved a sufficient pain therapy in pediatric patients with a NRS > 4 after trauma. In case of non-trauma, the pain management by the emergency physicians is restrained and less successful. The most common analgesic medications administered were ketamine and fentanyl, followed by piritramide.

*Trial registration*: The study has been retrospectively registered at DRKS (DRKS00026222).

## Background

The treatment of pain in pre-hospital pediatric emergencies continues to be a major challenge. In recent years, numerous studies have been conducted on this topic and the results have revealed that pre-hospital pain management in children is restrained and mostly inadequate [1–7]. Several best-practice guidelines have made recommendations for better pre-hospital pain management in children and recognized the need to make this a prioritized research topic [8–11]. The treatment of acute pain conditions is an elementary task of any emergency medical service (EMS). Pain therapy is particularly demanding in children because age, developmental stage, cognitive ability and communication skills must be taken into account [12]. Pre-hospitally, numerous measures are available to relieve pain, including non-pharmacological measures such as splinting, bandaging and cooling. If the pain is more severe, analgesia with medication is indicated; analgesics with varying degrees of effectiveness are always available for this purpose. Furthermore, many principles of pain management from existing recommendations for adults may also well apply to children. Inadequate pain management can have serious short and long-term consequences: Anxiety, fear of further medical treatment, post-traumatic stress disorder, development of chronic pain, and poorer recovery outcomes [13–16]. Several research groups have tried to identify risk factors for poor pain management in children: age < 5 years and short care time have been reported clustered causal factors for poor pain management [4, 17, 18]. Even in children with severe pain, it has been repeatedly shown that children have not received adequate pain management [6]. The reasons for these results are manifold; as recently reported also the lack of training and skills (e.g. iv line), dosage uncertainties, fears of adverse effects (e.g. bradypnea) and the missing routine caused by the rarity of pediatric emergencies seem to cause major problems [19, 20].

In Germany, there have been no studies on the quality of pain therapy in the pre-hospital period in childhood. However, a few studies from all over the world clearly show that there are deficits in pediatric pain therapy in the emergency medical service (EMS) and that this topic should be given more attention in scientific studies, especially in a physician staffed EMS.

The objective of this study was to evaluate the management of acute pain in children in the pre-hospital setting. This study analyzed the assessment of pain in children and the effectiveness of the analgetic treatment by emergency physicians with reference to etiology of pain and pain severity.

## Methods

A retrospective analysis of the German air rescue service *DRF Luftrettung (DRF, Filderstadt, Germany)* database was conducted. The database collects all operational data of the 29 DRF helicopters in Germany. The helicopters are alerted as part of the EMS additional to an ambulance manned by paramedics or even a rapid response car with physicians. They are care for child and adult emergencies alike. The medical crew on the rescue helicopter consists of an emergency physician (mostly specialists in anesthesiology, surgery or internal medicine) and a HEMS-TC, (helicopter emergency medical system technical crew member) moreover qualified as a paramedic. The physicians of the crew have mostly additional qualification in emergency care for children (such as European Pediatric Advanced Life Support (EPALS) course or more than 50 anesthetic procedures in children less than 5 years of age).

Each EMS mission is documented in a standardized online-database (HEMSDER-Database, Convexis, Germany). The collected data includes basic patient demographics as well as diagnostical and therapeutical actions of the emergency physician on scene. The severity of pain is assessed on a 0 (no pain) to 10 (maximum of pain) scale and was collected twice: First at arrival of the emergency physician on scene and second at admission to hospital.

Various analgesics are available on the helicopters, including the opioids fentanyl, sufentanil, piritramide and morphine. Ketamine and various non-opioid analgesics, like non-steroidal anti-inflammatory drugs (NSAID), are also available. All analgesics can be administered at the discretion of the emergency physician.

In the retrospective study period from 2012 to 2020 all emergency medical missions of DRF helicopters were included. Furthermore, we defined children with ages ranging from 0—16 years. The Glasgow Coma Scale (GCS) was used to distinguish between awake and anaesthetized patients. Patients with a GCS 3 at hospital admission were excluded from the study because a GCS3 is associated with general anesthesia, therefore we excluded these patients from the analysis. Furthermore, patients were excluded if the documentation was incomplete in relevant variables (e.g. missing vital signs). Based on the major diagnosis the cases were divided into a trauma and a non-trauma (e.g. acute abdomen) group. Patients with both, trauma and non-trauma, were excluded from further analyses. A value of > 4 in NRS was regarded as being moderate-to-severe pain and analyzed more in detail. The primary outcome measure was effective pain management, defined as a reduction of >  = 2 points on the NRS. Secondary outcomes were the analysis of the given pain medication.

For the descriptive analysis of numerical variables, the mean and standard deviation were computed, for categorial and dichotomous variables the frequency and proportion in percent were calculated. For comparative analysis Mann–Whitney-U or Chi-Quadrat-test (respectively Fisher´s exact test for small sample sizes) were used if applicable. Tests were two sided. A p-value of 0.05 or less was considered statistically significant. Calculations were performed with IBM SPSS Statistics 26 (Armonk, NY, USA).

The study was approved by the Ethics Committee of the Christian-Albrechts-Universität Kiel (registration: D 424/22) and registered in the German Register of Clinical Studies (Trial registration: DRKS 00,026,222).

## Results

### Study population

**Fig. 1:**
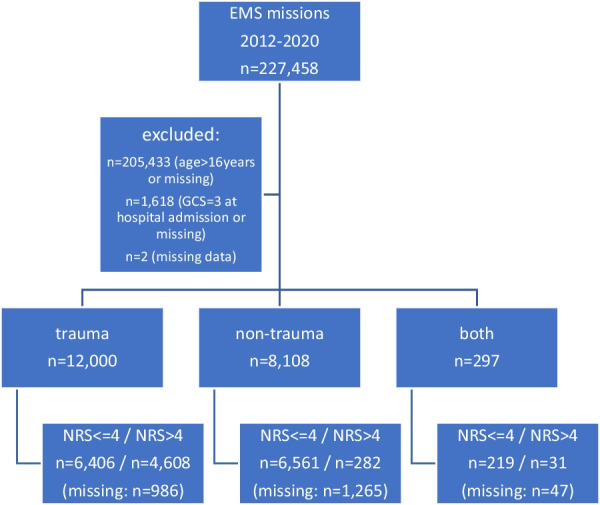
Flow chart of in- and exclusion criteria (CONSORT)

Out of 227,458 emergency missions in the study period, a total of 20,405 patients were included in the study (Fig. 1). Overall, 56.0% of the patients were male and 41.1% were female. No gender was reported in 2.9%. 12,000 patients suffered trauma, 8,108 had a non-trauma diagnosis as major diagnosis and 297 patients had both, a trauma and a non-trauma issue. Of the traumatized children 59.4% were male and 37.9% were female (missing 2.7%). Children with trauma were on average 8.2 (SD 5.2) years old. Children with a non-trauma were on average 6.2 (SD 5.6) years old. At arrival of the emergency physician on scene, 38.4% of the children in the trauma group and 3.5% in the non-trauma group had moderate to severe pain (NRS > 4). After the EMS therapy 2.0% in the trauma group and 0.6% in the non-trauma group had NRS > 4 at hospital admission (Table 1).

**Table 1:** General characteristics of the included cases

	Trauma	Non-trauma	Both
No. of cases	12,000	8,108	297
Age: mean (SD)	8.2 (5.2)	6.2 (5.6)	9.4 (5.5)
*Gender*			
Male	7125 (59.4%)	4146 (51.1%)	153 (51.5%)
Female	4549 (37.9%)	3707 (45.7%)	137 (46.1%)
Missing	326 (2.7%)	255 (3.1%)	7 (2.4%)
NRS at EMS arrival: mean (SD)	3.9 (2.9)	0.5 (1.5)	1.8 (2.2)
Missing	986	1265	47
NRS at EMS arrival > 4	4608 (38.4%)	282 (3.5%)	31 (10.4%)
Missing	986 (8.2%)	1,265 (15.6%)	47 (15.8%)
NRS at hospital admission: mean (SD)	1.5 (1.3)	0.3 (0.9)	1.0 (1.3)
Missing	969	1,139	38
NRS at hospital admission > 4	244 (2.0%)	51 (0.6%)	4 (1.3%)
Missing	969 (8.1%)	1,139 (14.0%)	38 (12.8%)
Development of pain during EMS treatment^*a^			
Pain reduction (= 1 scale point)	925 (7.7%)	144 (1.8%)	19 (6.4%)
Effective pain reduction (> = 2 scale point)	5501 (45.8%)	382 (4.7%)	48 (16.2%)
No change	4,424 (36.9%)	6,263 (77.2%)	179 (60.3%)
Increase of pain (> = 1 scale point)	86 (0.7%)	32 (0.4%)	3 (1.0%)
Missing	1064 (8.9%)	1287 (15.9%)	48 (16.2%)

^*^^a^Difference between NRS at EMS arrival and hospital admission

### NRS > 4, trauma

In 4,608 out of 12,000 children (38.4%) with trauma the initial NRS was > 4. The first measured mean NRS value in this group was 6.9 (SD 1.5). For analgesic treatment sufentanil und fentanyl were used in 40.8% (fentanyl 38.7% and sufentanil 2.2%) and piritramide in 19.9%. Morphine was given in 1.6%. Ketamine was administered in 34.5%. 3.1% of the patient received a non-steroidal anti-inflammatory drug (NSAID) mono therapy and 3.9% and NSAID combination therapy. A total of 9.4% within this group received no pain medication. After the EMS therapy, the mean NRS value was 1.9 (SD 1.4) at admission to hospital, 4.9% of the children still had an NRS > 4 (Table 2). An effective reduction in pain (> = 2 scale points) was achieved in 95.9% of children with trauma and initial NRS > 4. In 2.7% of the children there was no change in NRS until handover at the hospital.

**Table 2:** Patient with NRS > 4 after trauma or non-trauma

	Trauma	Non-trauma	Test	*p*-Value
No. of cases	4,608	282		
Age: mean (SD)	9.8 (SD 4.9)	11.4 (4.5)		
*Gender*				
Male	2789 (60.5%)	124 (44.0%)		
Female	1702 (36.9%)	152 (53.9%)		
Missing	117 (2.5%)	6 (2.1%)		
NRS at EMS arrival: mean (SD)	6.9 (1.5)	6.7 (1.5)	U	0.029
Missing	0	0		
NRS at EMS arrival > 4	4608 (100%)	282 (100%)		
Missing	0	0		
NRS at hospital admission: mean (SD)	1.9 (1.4)	2.7 (1.7)	U	*p* < 0.001
Missing	56	4		
NRS at hospital admission > 4	228 (4.9%)	45 (16.0%)	Chi^2^	*p* < 0.001
Missing	56 (1.2%)	4 (1.4%)		
Opioid therapy	2858 (62.0%)	101 (35.8%)	Chi^2^	*p* < 0.001
Therapy with strong opioids^*a^	1882 (40.8%)	46 (16.3%)		
Therapy with less strong opioid^*b^	976 (21.2%)	55 (19.5%)		
Ketamine	1590 (34.5%)	8 (2.8%)	Chi^2^	*p* < 0.001
*NSAID therapy*			Chi^2^	*p* < 0.001
No NSAID therapy	4283 (92.9%)	147 (52.1%)		
NSAID and opioid	181 (3.9%)	39 (13.8%)		
NSAID mono	144 (3.1%)	96 (34.0%)		
No pain medication	432 (9.4%)	80 (28.4%)	Chi^2^	*p* < 0.001
Development of pain during EMS treatment^*c^			Chi^2^	*p* < 0.001
Pain reduction (= 1 scale point)	60 (1.3%)	12 (4.3%)		
Effective pain reduction (> = 2 scale point)	4366 (95.9%)	240 (86.3%)		
No change	123 (2.7%)	25 (9.0%)		
Increase of pain (> = 1 scale point)	3 (0.1%)	1 (0.4%)		
Missing	56	4		

^*^^a^Includes fentanyl and sufentanil

^*^^b^Includes morphine, piritramide and other opiates

^*^^c^Difference between NRS at EMS arrival and hospital admission

**Fig. 2:**
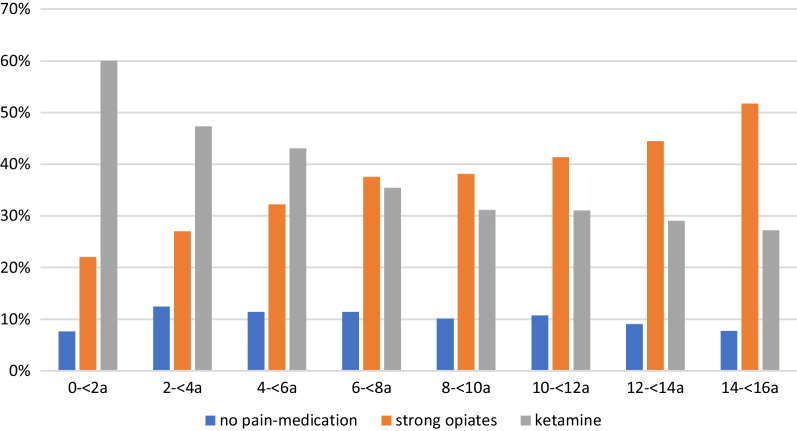
Pain medication in children with trauma and moderate to severe pain (NRS>4) at EMS arrival in age cohorts

The pain therapy in children with NRS > 4 after trauma differed between the observed age groups (Fig. 2. In the age group 0—< 2 years 22.0% received a strong opioid. The therapy with strong opioids increased to 32.2% in the age group of the 4- < 6 years and further to 51.7% of the adolescents between 14 – 16 years. Ketamine was used in younger patients aged 0 – < 2 years in 60.0% respectively. In the age group between 6—< 8 years, ketamine was still used in 35.4% and in adolescents in 27.2%. Non-opioid analgesics were used in less than 10% across all age groups.

Children treated with NSAID mono therapy or without pain medication had a mean NRS 6.2 at EMS arrival. NSAID mono therapy resulted in a percentage of 10.4% patients with relevant pain at hospital admission. Of the patients without any pain medication after a trauma with NRS > 4 on scene (n = 432), 15.7% reported pain (NRS > 4) at hospital admission.

### NRS > 4, non-trauma group

282 (3.5%) patients with pain in the non-trauma group had an NRS > 4 on scene (Table 2). The first measured mean NRS value in this group was 6.7 (SD 1.5) and 2.7 (SD 1.7) after arrival at the hospital. An effective reduction in pain (> = 2 scale points) was achieved in 86,3% of children in the non-trauma group and initial NRS > 4. In 9% of the children there was no change in NRS until handover at the hospital. For analgesic treatment strong opioids were administered in 16.3% (e.g. fentanyl), weaker opioids in 19.5% (e.g. piritramide, morphine), Ketamine was administered in 2.8%. Metamizole was administered in 41.5% and paracetamol in 4.3%. A total of 28.4% within this group received no pain medication. 16.0% of the children in the non-trauma group with NRS > 4 at EMS arrival still had an NRS > 4 at hospital admission.

## Discussion

This study evaluated 20,405 pediatric emergencies with 12,000 children being affected by trauma and 8,108 children with acute pain due to non-trauma. 4,608 (38.4%) children with trauma had a NRS > 4 at first survey (mean 6.9 (SD 1.5)). Children with acute pain due to non-trauma were less likely to have moderate to high pain scores (3.5%) but if relevant pain was present (NRS > 4), it tended to be severe (mean NRS 6.7 (SD 1.5)) (Table 1). Surprisingly, analgetic treatment differed significantly despite similar NRS regarding trauma and acute pain due to non-trauma. Thus, children in the trauma group received opioids in 62.0% and ketamine in 34.5%. In the non-trauma group opioids (35.8%) and ketamine (2.8%) were administered significantly less frequent and paracetamol and metamizole were given instead.

Over the past decade, numerous recommendations have been published on pre-hospital pain management in children [10], which have not yet found widespread implementation. Different drugs for analgesia have been suggested and recommended depending on the severity of the pain. But this also raises the question which analgesics are available in the different ambulance systems and how experienced the users are with possible application routes (e.g. intravascular, nasal or intramuscular). For management of moderate to severe pain, fentanyl appears to be the first line treatment for children [10, 28]. In our study, fentanyl was primarily used for analgesia, with piritramide being the second most commonly opioid used. The results of our study show a trend towards restrained use of opioids in young children and an increase in opioid administration in children older than 6 years, these results are consistent with a recent study by Rugg et al. [29]. In children with an initial NRS > 4, an opioid was administered in 62% of all cases. In toddlers and infants with severe pain, ketamine is the most commonly used analgesic. We assume that there is a greater uncertainty in the treatment of younger children, especially regarding the side effects of opioids. Thus, it indeed seems convincing to rather use ketamine due to its lower side effect profile and/or to apply opioids intranasally with the aim of achieving a greater therapeutic breadth [28, 30, 31]. From the age of 6 years, opioids are more often administered for analgesia and the use of ketamine becomes less frequent.

The comparison of the trauma and the non-trauma group showed a lower NRS in trauma children at the time of hospital admission (mean NRS 1.9) than in children with non-trauma (mean NRS 2.7). When NSAID mono therapy or even no analgesics were administered, the results were poor: 10.4% NSAID resp. 15.7% (no analgesics) of the patients reported NRS > 4 at hospital admission (Table 2). Around 9,4% of all children with trauma and a total of 28,4% in the non-trauma group with NRS > 4 received no pain medication at all.

We consider these numbers to be worrying because they clearly reflect uncertainty in the treatment of children. We can only speculate about the causes of the inadequate pain therapy in our setting, as all emergency physicians on our helicopters are required to have expertise in pediatric analgetic treatment. However, our findings are consistent with those of many other studies that have drawn attention to inadequate pain management for children in emergency medicine worldwide. Another study of pre-hospital analgesia found that although pain was noted in 446 cases, analgesia was administered in only 3.3% [2]. In one Canadian study, children with fractures of the extremities received analgesia in 37%, but only 3.2% received opioids [3]. Lord et. al also reported that in the case of the most severe pain (NRS 8–10), only 45% of children received analgesia [6]. According to these findings, children are at comparatively high risk for inadequate pre-hospital analgesia. A comparison of the studies is difficult as the ambulance systems in the different countries are not necessarily identical and a distinction must be made between physician and non-physician care. We believe that the more appropriate pain management in our study compared to most other studies is related to the fact that an emergency physician (trained in pediatric emergencies) was always involved.

Murphy et al. describe possible reasons such as communication problems in infants and young children, inadequate training of emergency personnel, and uncertainty and lack of experience in the assessment and care of children [21]. Paramedics and physicians are trained in providing and treating children, but pediatric emergencies occur less frequently, and invasive procedures and therapies are rarely needed. Reluctance associated with placing intravenous access in children appears to be a significant concern for paramedics and physicians, leading to hesitation and ultimately contributing to lower administration of analgesics [17].

The difficulty of correctly assessing pain in children has been cited as another potential cause of inadequate pain management in children. The major problem in pain assessment appears to be subjectivity and finding an adequate scale for the age. In our study, NRS was reported for almost all children, which is possibly due to the electronic patient report form used, in which two documented NRS scores are mandatory, one at first contact on scene and one at hospital admission. A large proportion of our patients were younger than 8 years, limiting the applicability of the NRS. Certainly, some children were unable to assign a number to their pain, e.g. depending on their common status. Presumably, the reported value in these patients corresponded to the personal impression of the emergency physician. It remains unclear whether in some cases other scores were additionally collected but not documented. In several studies the NRS was validated in children aged 8 + years [22]. The minimal clinically relevant difference in the numerical rating scale is 2 points on a 10- point scale [23, 24]. Other authors define effective analgesia as a reduction in pain score by at least 30% [25]. In our study population, an effective reduction in pain (> = 2 scale points) was achieved in 95.9% for pediatric trauma and resp. 86.3% for children in the non-trauma group (Table 3).

**Table 3:** Characteristics of patients with NRS > 4 after trauma depending on pain therapy

	Therapy with powerful opioids^*a^	Therapy with less powerful opioids^*b^	Ketamine and opioid	Ketamine without opioid	NSAR mono therapy	No pain medication
No. of cases	1604	838	416	1174	144	432
Age: mean (SD)	11.1 (4.3)	10.3 (4.4)	9.9 (5.0)	8.0 (5.2)	8.6 (5.5)	9.4 (4.7)
*Gender*						
Male	1,000 (62.3%)	495 (59.1%)	276 (66.3%)	703 (59.9%)	73 (50.7%)	242 (56.0%)
Female	563 (35.1%)	321 (38.3%)	134 (32.2%)	438 (37.3%)	68 (47.2%)	178 (41.2%)
Missing	41 (2.6%)	22 (2.6%)	6 (1.4%)	33 (2.8%)	3 (2.1%)	12 (2.8%)
NRS at EMS arrival: mean (SD)	7.0 (1.5)	6.6 (1.4)	7.5 (1.6)	7.2 (1.5)	6.2 (1.4)	6.2 (1.3)
Missing	0	0	0	0	0	0
NRS at hospital admission: mean (SD)	1.9 (1.3)	2.1 (1.3)	1.7 (1.3)	1.6 (1.4)	2.7 (1.6)	2.5 (1.7)
Missing	9	4	5	28	0	10
NRS at hospital admission > 4	63 (3.9%)	43 (5.2%)	10 (2.4%)	29 (2.5%)	15 (10.4%)	68 (15.7%)
Missing	9	4	5	28	0	10
Development of pain during EMS treatment*^c^						
Pain reduction (= 1 scale point)	9 (0.6%)	7 (0.8%)	1 (0.2%)	6 (0.5%)	10 (6.9%)	27 (6.4%)
Effective pain reduction (> = 2 scale point)	1,566 (98.2%)	805 (96.5%)	405 (98.5%)	1,124 (98.1%)	126 (87.5%)	340 (80.6%)
No change	20 (1.3%)	21 (2.5%)	4 (1.0%)	16 (1.4%)	8 (5.6%)	54 (12.8%)
Increase of pain (> = 1 scale point)	0	1 (0.1%)	1 (0.2%)	0	0	1 (0.2%)
Missing	9	4	5	28	0	10

^*^^a^Includes fentanyl and sufentanil without ketamine

^*^^b^Includes morphine, piritramide and other opioids without ketamine

^*^^c^Difference between NRS at EMS arrival and hospital admission

For children, there are further scales according to their age, including the Wong-Baker faces scale, on which children are asked to select the face that best represents their pain; this scale has been validated from the age of 3 years [26]. The Children´s Discomfort and Pain Scale according to Büttner is used for pain assessment in children from neonatal age to the age of four and has been validated for postoperative pain [27]. To our knowledge, there are no validated pain scoring systems for children in the pre-hospital setting and there is no requirement at DRF to use one specific scoring method. Nevertheless, we are convinced that the common scales are also suitable for the assessment of pain in the pre-hospital setting. Furthermore, the available data show that the pain scale used reliably reflects the treatment success.

### Limitations

Our study has several limitations starting with the retrospective character. As mentioned above, accuracy and objectiveness of the pain assessment might be a query. We were also not able to assess the influence of non-pharmacological pain management techniques such as slings and bandages. That is interesting because some patients with severe pain also showed improvement in NRS even though they did not receive analgesic medication. In these cases, conservative measures may have alleviated the pain. We were also not able to assess the influence of transport time on pain relief. Bendall et al. reported that children with a shorter care time are less likely to achieve effective pain management (18). Whitley et al. found no coherence concerning the length of transport to the hospital and effective pain management. Due to the digital data collection, it was not possible to record which dosages of each medication administered and the application route chosen.

## Conclusions

Overall, the results show that a sufficient pain therapy could be achieved especially in patients with NRS > 4. Children with trauma received more pain medication than in the context of non-trauma, even if the pain was comparably severe. In Germany the most common analgesic medications administered were ketamine and fentanyl, followed by piritramide. Ketamine was given more frequently to children under 6 years of age, while opioids were given more frequently with increasing age. To further improve sufficient pain management in children and reduce anxiety, dosing tools should be available in all settings and regular training with them should be provided.

## Data Availability

The data that supports the findings of this study are available from German air rescue service association (DRF e.V.) but restriction apply to the availability of this data, which were used under license for the current study, and so are not publicly available. Data are however available from the authors upon reasonable request and with permission of German air rescue service association (DRF e.V.).

## Abbreviations

DRF: Deutsche Rettungsflugwacht e.V.
EMS: Emergency medical service
EPALS: European pediatric advanced life support
GCS: Glasgow coma scale
HEMS-TC: Helicopter emergency medical system technical crew member
NRS: Numerical rating scale
NSAIDS: Non-steroidal anti-inflammatory drug
